# A systematic review and equity analysis of school-based violence prevention interventions evaluated in randomised controlled trials

**DOI:** 10.1186/s12889-025-26142-1

**Published:** 2026-01-28

**Authors:** Emily Eldred, Karen Devries, Kate A. Nelson, Anja Zinke-Allmang, Charles Opondo, Waliyah Mughis, Rizwana Mallick, Lena Morgon Banks, Amiya Bhatia

**Affiliations:** 1 Faculty of Epidemiology and Population Health, London School of Hygiene & Tropical Medicine, Keppel Street, London, WC1E 7HT UK; 2 Department of Social Policy and Intervention, University of Oxford, Oxford, UK; 3 Department of Community Health Sciences and the Brain & Mind Institute, Aga Khan University, Karachi, Pakistan; 4 Children’s Institute University of Cape Town, Cape Town, South Africa

**Keywords:** Violence prevention, Schools, Systematic review, Health inequities, Randomised controlled trials

## Abstract

**Background:**

Experiencing violence in school is a violation of a child’s rights and can increase school absence, affect academic achievement, employment prospects, and later-life health. Effective school-based violence prevention interventions are crucial, however, most are universally targeted. It is less clear if current interventions to prevent violence in schools ensure subgroups of children benefit equitably.

**Methods:**

We conducted a systematic review. We drew on professional networks and searched Medline, Cochrane Library, Embase, Global Health, PsycINFO, and Web of Science to identify systematic reviews (*n* = 29) which included randomised controlled trials (RCTs) of school-based violence prevention interventions. Reviews conducted searches until December 2023. We screened all included articles within the final review sample to identify all RCTs of universal school-based violence prevention interventions. We examined what sociodemographic characteristics were measured, assessed intervention effectiveness by subgroups, and applied criteria to assess equity.

**Results:**

Out of 160 articles of RCTs evaluating violence prevention interventions in schools, we identified 19 articles reporting on 16 trials that reported effects by the following subgroups: sex (*n* = 16), disability (*n* = 1), sexuality (*n* = 1), race/ethnicity (*n* = 1) and socioeconomic status (*n* = 1). Subgroup and moderation analysis found mixed results of intervention effectiveness by subgroups, with some evidence of heterogeneity.

**Conclusions:**

Most trials of school-based violence prevention interventions do not report whether they are effective for subgroups of minoritised children, or children at higher risk of experiencing violence, and therefore may not contribute to advancing health equity. Ensuring a commitment to equity in the design, delivery, and impact of school-based violence prevention is essential to achieving the Sustainable Development Goals.

**Review registration:**

PROSPERO: CRD42023463384.

**Supplementary Information:**

The online version contains supplementary material available at 10.1186/s12889-025-26142-1.

## Background

Across the world, 1 billion children experience violence every year [1], which often takes place in schools [2–5]. Prior evidence suggests that experiences of violence are not equally distributed between children. The frequency and severity of violence victimisation is often greater amongst minoritised children who often unfairly experience structural and social disadvantage or social stigma [6–11].

Evidence of social disadvantage as a structural determinant of violence is often seen in school settings. For example, children with disabilities are twice as likely to experience violence compared to children without disabilities [12], including more violence within schools [13]. Several studies examining peer and dating violence have found that sexual minority students experience higher levels of victimisation than heterosexual students [14–18]. Levels of violence are disproportionately high for trans children [18–22]. A recent review found racial and ethnic minorities, immigrants, and refugee children have a higher risk of experiencing bullying in school environments that were discriminatory or harbored negative stereotypes [23]. The uneven distribution violence among children is preventable, unjust and unfair, and constitutes a health inequity [24–26].

Schools are formative social environments for children and support cognitive development, relationship-building, and critical thinking [27]. Schools can therefore be an important platform for behavioural change interventions aiming to prevent violence [28–34]. While it is likely that most school-going children, including minoritised children, are present in schools where violence prevention interventions are implemented, it is less clear the extent to which interventions and their evaluations are adequately inclusive or are effective for subgroups of children who are more likely to experience violence victimisation. Further, evaluations of violence prevention interventions do not always consider if interventions increase inequities by benefitting children that may be better off [35].

Guidance on designing and reporting on equity considerations in trials is increasing, including the CONSORT-equity trial reporting guidelines, PROGRESS-PLUS outlining individual and social characteristics for measurement, and REP-EQUITY on recruiting an equitable sample [36–40]. Although these developments over the past 10 years indicate progress on developing an evidence base that contributes to health equity in a population, these guidelines are often not applied in RCTs of school-based violence prevention interventions.

In this systematic review we take an equity lens to first assess the extent to which minoritised children are included in universal school-based violence prevention intervention evaluations and identify which evaluations examined intervention effects among subgroups. Among evaluations that included subgroups, we then conduct an equity assessment to describe quality of inclusion and assess whether school-based violence prevention interventions are effective amongst different groups of children and/or have differential effects by subgroups.

## Methods

### Searches and screening

This paper is part of a systematic review registered on PROSPERO (CRD42023463384). We outline further details of the searches and screening elsewhere [41]. Briefly, the screening was conducted in three stages. First, in July 2023 we searched for systematic reviews of school-based violence prevention interventions (see Appendix 5 for search terms). Records that included key terms in the title or abstract were retrieved from: Medline, Cochrane Library, Embase, Global Health, PsycINFO, and Web of Science. In May 2024 we included a further two reviews identified via professional networks [42]. One of these reviews was unpublished and included records up to December 2023.

Second, we screened the papers within the identified systematic reviews with the following inclusion criteria: (a) randomised controlled trials (RCTs); (b) school-based interventions; (c) violence outcome(s); (d) outcomes measured among nursery, primary or secondary school children. We excluded theses, conference proceedings, and books. We applied no restrictions to publication date. At stage 1 and 2, EE completed the screening, with 10% of papers double screened by AZA.

In the third stage, we identified studies that reported subgroup-specific effect estimates or tests of heterogeneity by different sociodemographic characteristics associated with social disadvantage or higher risk of violence victimisation [43]. For these studies we restricted this sample further to include only papers that assessed self-reported violence victimisation outcomes, excluding outcomes that were perpetration, knowledge, or attitude focused. Trials with effects measured among single-group populations only (without a comparator to another group of children) were excluded, noting that while we consider trials targeted at single-groups of disadvantaged populations only (with no comparator) to be equity-relevant, this is not the focus of this review. We excluded studies that did not report empirical results, results without confidence intervals or only narrative descriptions of subgroup effects.

### Extraction

For all papers identified in stage 2, we extracted information on sociodemographic characteristics. Sociodemographic characteristics were initially defined using PROGRESS-PLUS and revised throughout data extraction [40, 44]. Data was extracted from each manuscript and appendices were only checked where referenced in the main text.

For our final sample of papers identified in stage 3, where data on effectiveness among subgroups were available, we extracted further information on the effect estimates (e.g. factors adjusted for, measure of effect, scale of interaction). If studies reported results from more than two time points, we defined and extracted the endline estimate that was specified a priori or in a protocol by the authorship team. If unspecified, we use the time point immediately after implementation. Data extraction was conducted by EE and final papers were double screened by KAN and CO.

### Equity assessment

We conducted an equity assessment of papers identified in stage 3. To assess equity within the trial design (Table 1, Appendix 2), we developed a framework from existing equity checklists, including CONSORT-Equity, REP-EQUITY and PROGRESS-PLUS, and best practice subgroup analysis guidelines [37, 38, 43, 45–48]. The framework was refined using a deductive approach. The final equity framework included 8 criteria that assessed the design of data collection (e.g. in sampling, consenting, referrals), analysis methodology (e.g. powering trials and analysis approach) and intervention design (e.g. targeting of intervention content and accessibility). Scoring criteria were assigned to each equity item to give each study an overall score from ‘none reported’ (0 items), ‘low’ (1–2 items), ‘medium’ (3–5 items), or ‘high’ (6–8 items). Where there was more than one publication per trial, we applied the equity criteria to each article and combined the total score for the trial. EE conducted the assessment with 10% double screened by WM and discrepancies were discussed in meetings between the reviewers.

**Table 1:** Equity criteria for assessing trial design of school-based violence prevention trials that conducted a subgroup analysis*

No.	Criteria	Explanation of scoring (0 or 1)
1	Is the research question or hypothesis concerned with measuring equity or differences between groups?	Studies were given a score of 1 if they met any of the following criteria if the research question or hypothesis: a) Included any of the following terms in relation to two subgroups: equity, inequity, inequality, difference. or b) Included testing intervention effects between two subgroups with a hypothesis about which group would benefit more/less from the intervention specified a priori
2	Was the subgroup analysis reported to be adequately powered?	Studies were given a score of 1 if: a) authors describe/state that the study was powered to detect differences for at least one subgroup
3	Did the paper report if subgroup analysis were pre-specified?	Studies were given a score of 1 if the paper mentioned if: a) subgroup analysis were specified a priori
4	Was the method of analysing and reporting the sub-group results appropriate?	Studies were given a score of 1 if the method of analysing the sub-group was appropriate, based on: a) reporting of a sub-group specific effect estimate using an interaction term and corresponding test for heterogeneity
5	Was equity considered in sampling?	Studies were given a score of 1 if the methods section included a description or reference to any of the following: a) oversampling, or b) stratified sampling, or c) any other effort including for example, including a variation in the range of school sites (rural vs. urban)
6	Was equity considered in the design of survey implementation?	Studies were given a score of 1 if they included a description or reference to any of the following: a) adaptations to informed consent procedures (e.g. witness, thumbprint), or b) consent procedures for parents included oral explanations of study and reimbursement for travel costs, or c) adjustments to the survey delivery and questions (e.g. multiple languages were used to administer the survey that were relevant to the population), or d) training was provided to researchers on reasonable adjustments (e.g. for disability), or e) measures about inequities were included in the survey (e.g. measurement of gender equity), or f) any other similar adaptations made, in discussion with co-authors
7	Was equity considered in study response or referral plans?	An inclusive referral plan for cases of disclosures of violence is essential to violence research [49]. Studies were given a score of 1 if they included a description or reference to any of the following: a) adaptations to procedures for minoritised groups that are context specific. For example, children with disabilities (e.g. consultation with social workers about capacity for receiving disclosures of children with different impairments, including sign language)
8	Was there any adaptation to intervention content for subgroups?	We considered an adaptation to be a flexibility in the content and delivery to accommodate differences in the population. Studies were given a score of 1 if they included a description or reference to any of the following: a) any form of adaptation to intervention materials (e.g. audio-visual, braille), teachers or implementers trained in reasonable adjustments, or b) representative intervention material pictures and stories, or c) testing of intervention in different sociodemographic groups, or d) different languages used in intervention materials, or e) any other similar adaptations made to improve access to and/or impact of intervention for subgroups, in discussion with co-authors

* To develop criteria, we drew on existing reporting checklists, including CONSORT-Equity, REP-EQUITY and PROGRESS-PLUS, international guidelines on subgroup analysis, and expertise within the authorship team, and adapted these for school-based violence prevention trials [37, 38, 45–48]. Further details can be found in Appendix 2

### Quality assessment

We assessed each paper included at stage 3 for risk of bias. We used the Cochrane Risk of Bias tool and rated the risk of bias to be ‘low risk’, ‘some concerns’, or ‘high risk’. An overall score was given based on the sum of each domain and results are visualised using Risk-of-bias VISualization (robvis) tool [50]. EE conducted the assessment with 10% double screened by KAN.

### Data analysis

We described the sociodemographic characteristics reported in included studies at stage 2.

For studies included at stage 3, we described the results of the equity assessment. For studies that estimated a subgroup-specific effect using an interaction term and/or corresponding test for heterogeneity, we conducted a meta-synthesis following guidance from Cochrane Handbook [46] and described in a table. For the meta-synthesis we used primary violence victimisation outcomes. If primary outcomes were either not specified or not a self-reported victimisation outcome, we either described all secondary victimisation outcomes (if 2 or less) or described an overall measure of violence (e.g. any violence experienced from teachers and peers).

Our review follows reporting guidance on reporting equity-focused systematic reviews where applicable [45].

## Results

The initial search to identify systematic reviews of school-based violence prevention interventions yielded a total of 5,214 articles, after duplicates were removed (Appendix 1). Following the title and abstract screening and full text screening, a total of 29 systematic reviews were identified [28–34, 42, 51–70]. From the included systematic reviews, 511 articles were identified for further full-text screening, excluding duplicates. After applying our inclusion and exclusion criteria, we identified a total of 160 articles reporting RCT results of school-based interventions targeting different violence prevention outcomes in stage 2. We restricted the sample to violence victimisation outcomes only and identified a total of 88 articles. We then identified a total of 19 articles reporting results of 16 trials that included a subgroup specific effect estimate of a violence victimisation outcome in stage 3. We note that if two or more articles reported on the same trial (e.g. reporting different outcomes or different subgroup results) we counted these as 1 trial record. Studies were published between 1985 and 2023.

### Characteristics of all school-based violence prevention interventions

Across all 160 articles, 17 sociodemographic characteristics were measured (Table 2). Most studies measured and reported on different sociodemographic characteristics (ranging from 0 to 8). Sex of participants was most commonly measured. For the total sample of 160 articles, gender identity (4%) and parents’ housing (3%) were least commonly measured. Of the 88 articles including violence victimisation outcomes, religion (1%) was the sociodemographic characteristic least commonly measured. Of the final sample of 19 studies with subgroup analysis, gender identity, immigration status, and parents alive or orphan status were not measured at all.

**Table 2:** Characteristics of all school-based violence prevention interventions and participant sociodemographic characteristics measured

Study Characteristic	Sociodemographic characteristics measured in total number of studies evaluating school violence prevention (*n* = 160)		Sociodemographic characteristics measured in total number of studies evaluating school violence prevention with victimisation outcomes (*n* = 88)		Sociodemographic characteristics measured in total number of studies with subgroup analysis (*n* = 19)	
	*N* studies	% of studies	*N* studies	% studies	*N* studies	% studies
**Country**						
Low-middle income country (LMIC)	32	20*·*0	19	21*·*6	7	37
High income country (HIC)	128	80*·*0	69	78*·*4	12	63
**Violence outcome**						
Teacher violence	6	3*·*8	5	5*·*7	2	11
Cyberbullying	12	8*·*0	8	9*·*0	3	16
Bullying	47	29*·*4	35	39*·*7	8	42
Sexual violence	32	20*·*0	8	9*·*0	1	5
IPV/Dating violence*	33	20*·*6	20	22*·*7	4	21
Violent behaviour	10	6*·*3	1	1*·*1	0	0
Gender-based violence	4	3*·*0	1	1*·*1	1	5
Child marriage	2	1*·*3	0	0	0	0
Multiple	14	8*·*8	10	11*·*4	0	0
**School Type**						
Nursery/pre-school	2	1*·*3	0	0	0	0
Primary	61	38*·*1	31	35*·*2	5	26
Secondary	88	55*·*0	53	60*·*2	12	63
Mixed	9	6*·*0	4	4*·*5	2	11
**Sociodemographic characteristics reported in paper** ^†^						
Disability^‡^	13	8*·*1	5	5*·*7	3	16
Ethnicity	23	14*·*4	17	19*·*3	3	16
Race	9	5*·*6	5	5*·*7	0	0
Race/ Ethnicity	59	36*·*9	29	33*·*0	6	32
Sexuality	9	5*·*6	8	9*·*0	3	16
Sex	152	95*·*0	83	94*·*3	18	95
Gender identity^§^	4	2*·*5	2	2*·*3	0	0
Socioeconomic status^¶^	51	31*·*9	27	30*·*7	8	42
Religion	5	3*·*1	1	1*·*1	1	5
Parents alive/orphaned	5	3*·*1	3	3*·*4	0	0
Family structure**	28	17*·*5	17	19*·*3	2	11
Parents’ education	19	11*·*9	8	9*·*0	1	5
Parents’ employment	11	6*·*9	6	6*·*8	1	5
Parents’ housing	3	1*·*9	2	2*·*3	1	5
Second language	10	6*·*3	5	5*·*7	1	5
Social class or caste	5	3*·*1	3	3*·*4	1	5
Immigration status	12	7*·*5	5	5*·*7	0	0

Includes sociodemographic characteristics of individual students only (not at school level)

* includes sexual violence from an intimate partner

† includes all sociodemographic characteristics that apply in each paper, so percentages do not equal 100

‡ includes disability, functional difficulties, special schools or special education lessons

§ we considered articles to include ‘gender identity’ as opposed to sex where authors include options for students to either identify as non-binary or ask students to ‘self-describe’

¶ proxy measures of socioeconomic status were included, e.g. number of meals eaten; asset index; free school meals; below income threshold; perceived economic status; and family affluence

** this includes marital status of parents or living situation (e.g. lives with one parent)

### Equity analysis of evaluations that included subgroup specific effect estimates

There were 19 articles, reporting on 16 trials, that reported a subgroup specific effect estimate. Details are outlined in Table 3. Using our equity criteria (Table 1), 2/16 trials had an equity hypothesis or research question, 4/16 were powered for subgroup analysis, none pre-specified subgroup analysis, 3/16 used appropriate methods to conduct an equity analysis, 5/16 considered equity in sampling, none considered equity in referral planning, 6/16 considered equity in implementation and 6/16 adapted intervention content to subgroups. For the overall scoring based on the sum of each equity criteria, 6 met none of the criteria, 5 had a low score (1–2 points), 5 had a medium score (3–5 points) and none had a high score (6–8 points).

Overall, the risk of bias (RoB) was rated as ‘some concerns’ for all studies. The domain ‘bias in measurement of the outcome’ was rated as ‘some concerns’ across all the trials and ‘bias due to deviations from the intended intervention’ also scored ‘some concerns’ in 9 trials due to the difficulty of masking behavioural interventions in the intervention schools and subsequent social desirability bias in participant reporting on the outcomes. Across the other domains, the rating of ‘some concerns’ related to no publicly available statistical analysis plan, trial registration or protocol, and missing outcome data. Further details can be found in appendix 4.

**Table 3:** Equity analysis of school-based violence prevention trials that conducted a sub-group analysis

Sociodemographic characteristic	Subgroup specific estimates (*n* = 16 trials^†^)		Equity hypothesis or RQ	Powered for sub-group analysis	Sub-group analysis specified a priori	Appropriate sub-group analysis	Equity considered in sampling	Equity considered in survey implementation design	Equity considered in referral/response plan	Adaptations to intervention content	Overall assessment
	*n*	%									
**Sex**	12	84.2%	1/12	3/12	0/12	1/12	3/12	5/12	0/12	5/12	None reported: 5/12 Low: 3/12 Medium: 4/12
**Sex and sexuality**	1	5.3%	0/1	0/1	0/1	0/1	0/1	0/1	0/1	0/1	None reported: 1/1
Sex and disability*	1	5.3%	1/1	1/1	0/1	1/1	1/1	1/1	0/1	0/1	Medium: 1/1
**Sex and socioeconomic status**	1	5.3%	0/1	0/1	0/1	1/1	1/1	0/1	0/1	0/1	Low: 1/1
**Sex and race/ethnicity**	1	5.3%	0/1	0/1	0/1	0/1	0/1	0/1	0/1	1/1	Low 1/1

Includes demographic characteristics of students only (not schools). Includes victimisation outcomes only. Includes reporting from trial paper only. Sociodemographic characteristics are grouped if papers reported more than one subgroup analysis

* disability includes descriptions of disability, functional difficulties, special education needs classification

† as we are interested in trial design, here we report on 16 trials reported in 20 papers. We conducted assessments for each paper and combined scores for each trial

### Equity of intervention effects

10/16 trials found evidence for an overall effect of the intervention, with the remaining finding no evidence of an overall effect or not including a measure for overall effect.

Subgroup estimates were reported across 5 sociodemographic characteristics, which included all trials estimating by sex (*n* = 16) and estimates by disability (*n* = 1), race/ethnicity (*n* = 1), sexuality (*n* = 1) and socioeconomic status (*n* = 1). Noting, several of these estimates were reported in the same paper. Among the 16 trials reporting intervention effectiveness by sex, 11 trials found evidence for an intervention effect in girls and 7 in boys for at least one victimisation outcome. The trial that reported intervention effectiveness by disability found evidence of an effect in children with disabilities and without disabilities with no evidence of a differential effect [13]. The trial that reported intervention effectiveness by sexuality found no evidence of an intervention effect in sexual minority groups but evidence of an effect for sexual majority students [71]. The trial that reported intervention effectiveness by race/ethnicity found evidence of an effect in African American children for physical violence but not for emotional violence and evidence of an effect for Hispanic children in emotional violence but not in physical violence [72]. The trial that reported intervention effectiveness by socioeconomic status found evidence of an effect in low and high socioeconomic status participants, with no evidence of a differential effect [73].

Notably, only 3 trials reported subgroup-specific effect estimates using an interaction term and corresponding test for heterogeneity in line with the Cochrane handbook [46]. Of these, 1 trial found the intervention to be more effective in girls, 1 trial found the intervention to be more effective in boys, and 1 trial found no evidence of differential effects.

All 19 articles reporting on 16 trials are summarised in Table 4 and further details can be found in Appendix 3.

**Table 4:** Subgroup-specific effect estimates in school-based violence prevention trials

**Effect estimates by sex**						
***Study (intervention; citation; violence outcome)***	***Overall intervention effect (average effect across all subgroups)***	***Risk of bias assessment***	Subgroup-specific effect estimate reported*		**Was there a differential effect by subgroup?** ***p*** **-value from test for heterogeneity**	***Interpretation of findings***
			***Effect in girls***	***Effect in boys***		
* Gender Equity Movement in Schools – GEMS in Viet Nam (Achyut*,* 2017)* ***Outcomes***: *any violence victimisation by peer in the last semester; any violence victimisation by teachers in the last semester*	*Peer violence*: *effect* ^*†*^ *= -2·50* *Teacher violence*: *effect = 2·70*	*Some concerns*	*Peer violence*: *effect= -1·40*	*Peer violence*: *effect= -3·20*	*Not reported*	*The intervention was not found to be effective overall*,* or for girls and boys*
			*Teacher violence*: *effect= -1·50*	*Teacher violence*: *effect = 8·30*		
* Gender Equity Movement in Schools – GEMS in India (Achyut*,* 2017)* ***Outcome***: *any violence victimisation by teacher or peer in the last 3 months*	***effect = 9·40,*** ***p*** ***< 0·01***	*Some concerns*	***effect = 14·70***	*effect = 2·90*	*Not reported*	*The intervention was found to be effective overall and for girls*,* but not for boys*
* Learning Together (Bonell*,* 2018)* ***Outcome***: *bullying victimisation in the past 3 months*	***Adjusted mean difference= − 0·03, − 0·06 to − 0·00 (95% CI); adjusted effect size − 0·08, p= 0.04***	*Some concerns*	***-0·03***,***-0·06 to 0·01 (95% CI)***	***-0·04, -0·08 to 0·00 (95% CI)***	*No* (*p* = 0·61)	*The intervention was found to be effective overall and for boys and girls. The study found no evidence for a difference in effect by sex*
* Fourth R (Cissner*,* 2014)* ***Outcome***: *dating violence victimisation; peer victimisation; sexual harassment/assault victimisation*	*Dating violence*: *SMD=-0·01* *Peer victimisation: SMD = 0·10* *Sexual assault*: *SMD = 0·07*	*Some concerns*	*Not reported*	*Not reported*	*No (p-value > 0.10 but not reported)*	*There was no evidence the intervention was effective for the three victimisation outcomes. There was some evidence for a differential effect by sex on dating violence victimisation at T2 but not at T1*
* Green Dot (Coker*,* 2017)* ***Outcome***: *sexual violence victimisation (all items)*	***PRR = 0·88***,*** 0·78 to 1·00 (95% CI)***	*Some concerns*	***PRR = 0·86, 0·74 to 1·00 (95% CI)***	*PRR = 0·91*,* 0·75 to 1·12 (95% CI)*	*Not reported*	*There was evidence of an effect overall. There was evidence of an effect for girls*,* but not for boys*
* Good Schools Toolkit (Devries*,* 2017)* ***Outcome***: *any violence from teachers or school staff in past week*	***aOR = 0·41, 0·26 to 0·64 (95% CI), p < 0·00***	*Some concerns*	***aOR = 0·49***,*** 0·31 to 0·77 (95% CI)***	***aOR = 0·34, 0·21 to 0·54 (95% CI)***	*Yes* (*p* = 0·00)	*The intervention was found to be effective overall. The intervention was effective for boys and girls*,* but more effective for boys*
* ViSC Social Competence Program (Gradinger*,* 2015)* ***Outcome***: *cyberbullying victimisation in the past 2 months*	***latent d = 0·29***	*Some concerns*	***d = 0·29***	*d = 0·29*	*Not reported*	*The intervention was found to be effective overall. The study found evidence for an effect in girls but not in boys*
* #Tamojunto (Gusmoes*,* 2018)* ***Outcome***: *physical violence victimisation in the past 30 days; bullying victimisation in the past 30 days*	*No measure of overall effect*	*Some concerns*	*Physical violence*,* aged 11–12*: *aOR = 0·88*,* 0·46 to 1·67 (95% CI)*	*Physical violence*,* aged 11–12*: *aOR = 0·87*,* 0·53 to1·42 (95% CI)*	*Not reported*	*The intervention was not found to be effective for girls or boys*,* except for the outcome of bullying for girls (aged 13–15) at 9 months* ^***‡***^
			*Physical violence*,* aged 13–15*: *aOR = 0·89*,* 0·42 to 1·86 (95% CI)*	*Physical violence*,* aged 13–15*: *aOR = 1·27*,* 0·74 to 2·18 (95% CI)*		
			*Bullying*,* aged 11–12*: *aOR = 0·93*,* 0·71 to 1·23 (95% CI)*	*Bullying*,* aged 11–12*: *aOR = 0·81*,*0·59 to 1·10 (95% CI)*		
			***Bullying, aged 13–15:*** ***aOR = 0·59***,*** 0·42 to 0·84 (95% CI)***	*Bullying*,* aged 13–15*: *aOR = 0·91*,* 0·64 to 1·28 (95% CI)*		
* Let Us Protect Our Future intervention (Jemmott*,* 2018)* ***Outcome***: *Experiences of forced sex*	*RR = 0·98*,* 0·96 to 1·00 (95% CI)*, *p* = 0.11	*Some concerns*	*Not reported*	*Not reported*	*Yes* (*p* = 0·02)	*There was no overall effect of the intervention. The intervention was more effective among boys than girls*
* KiVa (Karna*,* 2011)* ***Outcome***: *bullying victimisation*	***B= -0·15*** (***p < 0·00)***	*Some concerns*	*Not reported*	*Not reported*	*No (p-value > 0.1 but not reported)*	*The intervention was found to be effective. There was no evidence for a differential effect by sex*
* KiVa (Karna*,* 2013)* ***Outcome***: *bullying victimisation in the past 2 months in grades 2–3*^*§*^	***b= -0·49*** (***p < 0·01)***	*Some concerns*	***OR = 1·63, 1·34 to 1·91 (95% CI)***	***OR = 1·04, 0·79 to 1·30 (95% CI)***	*Yes* (*p* < 0·05)	*The intervention was found to be effective. The intervention was more effective among girls than boys*
* KiVa (Williford*,* 2013)* ***Outcome***: *cyberbullying victimisation in the past few months*	***OR = 1·29, 1·05 to 1·57 (p*** ***< 0·01)***	*Some concerns*	*Not reported*	*Not reported*	*No* (*p* = 0·37)	*The intervention was found to be effective. There was no evidence for differential effects by sex*
* Right to Play (Karmaliani*,* 2020)* ***Outcome***: *peer victimisation in the last 4 weeks*	*No measure of overall effect*	*Some concerns*	***EMD= -1·98***,*** -2·95 to -1·02***	***EMD= -1·57*** ***, -2·56 to -0·58***	*Not reported*	*The intervention was found to be effective in girls and boys*
* Early Childhood Friendship Project (Ostrov*,* 2015)* ***Outcomes***: *physical bullying victimisation; relational victimisation*	*Physical bullying*: *F(1*,* 101) = 1·78*, *p* = 0·19 ***Relational victimisation*** ***:*** ***F(1, 100) = 7·03, p = 0·00***	*Some concerns*	***Physical bullying*** ***:*** ***d= -0·74***	*Physical bullying*: *d = 0·11*	*Not reported*	*There was evidence of an overall effect for relational victimisation. There was evidence of an effect for girls for both outcomes*,* but not for boys*
			***Relational victimisation*** ***:*** ***d= -0·69***	*Relational victimisation*: *d = 0·04*		
* It’s Your Game…Keep It Real Program (Peskin*,* 2014)* ***Outcomes***: *physical dating violence victimisation; emotional dating violence victimisation*	***Physical victimisation:*** ***aOR = 1·52*** ***, 1·20 to 1·92 (95% CI)*** ***Emotional victimisation*** ***aOR = 1·74, 1·36 to 2·24 (95% CI)***	*Some concerns*	***Physical victimisation*** ***:*** ***aOR = 1·39*** ***, 1·05 to 1·84 (95% CI)***	***Physical victimisation*** ***:*** ***aOR = 1·84*** ***, 1·2 to 2·74 (95% CI)***	*Not reported*	*There was evidence of an overall effect*,* and an effect in girls and boys for all outcomes*
			***Emotional victimisation*** ***:*** ***aOR = 2·03*** ***, 1·44 to 2·84 (95% CI)***	***Emotional victimisation*** ***:*** ***aOR = 1·47*** ***, 1·06 to 2·04 (95% CI)***		
* SEHER (Shinde 2018)* ***Outcome***: *Violence victimisation in the past 6 months*	***SM vs. Control*** ^¶^ ***aOR = 0·62*** ***, 0·46 to 0·84 (95% CI)***	*Some concerns*	***SM vs. Control*** ***aOR = 0·57*** ***, 0·31 to 1·02***	***SM vs. Control*** ***aOR = 0·65*** ***, 0·46 to 0·90***	*Yes* (*p* = 0·02)	*The intervention was found to be effective overall in the SM group but not the TSM group. The intervention was more effective amongst girls than boys in both the SM and TSM groups*
	*TSM vs. Control* *aOR = 1·27*,* 0·93 to 1·73 (95% CI)*		*TSM vs. Control* *aOR = 1·75*,* 0·97 to 3·14*	*TSM vs. Control* *aOR = 1·16*,* 0·83 to 1·62*	*Yes* (*p* = 0·00)	
* Tabby Improved Prevention and Intervention Program (Sorrentino*,* 2018)* ***Outcome***: *cyberbullying victimisation in the past 6 months*	***F(1***,***620) = 5·23;*** ***p = 0·02)***	*Some concerns*	*F(1*,*1000) = 1·28*	***F(1***,***1000) = 10·68***	*Not reported*	*The intervention was found to be effective overall*,* and for boys. The intervention was not found to be effective amongst girls*
* GV/SH Prevention Programming (Taylor 2010)* ***Outcome***: *any peer victimisation; any dating victimisation*	Interaction-based treatment**: *SMD= -0·04 (SE = 0·11) (peer)* *SMD = 0·06 (SE = 0·06) (dating)* *Law and justice treatment*: *SMD = 0·01 (SE = 0·10) (peer)* *SMD = 0·02 (SE = 0·55) (dating)*	*Some concerns*	*Not reported*	*Not reported*	*No (p-value > 0.1 but not reported)*	*The intervention was not found to be effective. There were no differences by sex*
***Effect estimates by disability status***						
***Study (intervention; citation; violence outcome)***	***Overall intervention effect (average effect across all subgroups)***	***Risk of bias assessment***	Subgroup-specific effect estimate reported*		**Was there a differential effect by subgroup?** ***p*-value from test for heterogeneity**	***Interpretation of findings***
			***Effect in children with disabilities***	***Effect in children without disabilities***		
* Good Schools Toolkit (Devries*,* 2018)* ***Outcome***: *any violence from teachers or school staff in past week*	***aOR = 0·40, 0·26 to 0·64 (95% CI), p < 0.00***	*Some concerns*	***aOR = 0·27*** **,** *** 0·13 to 0·56 (95% CI)***	***aOR = 0·43*** ***, 0·27 to 0·67 (95% CI)***	*No* (*p* = 0·34)	*The intervention was found to be effective overall. There was no evidence of a difference in effect by disability status*
***Effect estimates by sexuality***						
***Study (intervention; citation; violence outcome)***	***Overall intervention effect (average effect across all subgroups)***	***Risk of bias assessment***	Subgroup-specific effect estimate reported*		**Was there a differential effect by subgroup?** ***p*-value from test for heterogeneity**	***Interpretation of findings***
			**Effect in sexual minority students**	**Effect in sexual majority students**		
* Green Dot (Coker 2020)* ***Outcome***: *any sexual violence victimisation*	***aPRR = 0·87, 0·77 to 0·99 (95% CI)***	*Some concerns*	*aPRR = 0·99*,* 0·83 to 1·19 (95% CI)*	***aPRR = 0·83*** ***, 0·71 to 0·99 (95% CI)***	*Not reported*	*There was evidence of an overall effect. There was no evidence of an effect for sexual minority students but there was evidence of an effect for sexual majority students*
***Effect estimates by race/ethnicity***						
***Study (intervention; citation; violence outcome)***	***Overall intervention effect (average effect across all subgroups)***	***Risk of bias assessment***	Subgroup-specific effect estimate reported*		**Was there a differential effect by subgroup?** ***p*-value from test for heterogeneity**	***Interpretation of findings***
			**Effect in African American children **	**Effect in Hispanic children **		
* It’s Your Game…Keep It Real Program (Peskin*,* 2014)* ***Outcomes***: *physical dating violence victimisation; emotional dating violence victimisation*	***Physical victimisation:*** ***aOR = 1·52, 1·20 to 1·92 (95% CI)*** ***Emotional victimisation:*** ***aOR = 1·74, 1·36 to 2·24 (95% CI)***	*Some concerns*	***Physical victimisation:*** ***aOR = 1·65*** **,** ***1·19 to 2·28 (95% CI)***	*Physical victimisation*: *aOR = 1·21*,* 0·71 to 2·04 (95% CI)*	*Not reported*	*There was evidence of an effect for both outcomes overall. There were mixed results for Hispanic and African American students*
			*Emotional victimisation*: *aOR = 1·70*,* 0·94 to 3·07 (95% CI)*	***Emotional victimisation*** ***:*** ***aOR = 1·78*** ***, 1·22 to 2·60 (95% CI)***		
***Effect estimates by socioeconomic status***						
***Study (intervention; citation; violence outcome)***	***Overall intervention effect (average effect across all subgroups)***	***Risk of bias assessment***	Subgroup-specific effect estimate reported*		**Was there a differential effect by subgroup?** ***p*-value from test for heterogeneity**	***Interpretation of findings***
			**Effect in low socioeconomic status**	**Effect in high socioeconomic status**		
* Learning Together (Bonell*,* 2018)* ***Outcome***: *bullying victimisation in the past 3 months*	***Adjusted mean difference= − 0·03, − 0·06 to − 0·00 (95% CI); adjusted effect size − 0·08, p = 0.04***	*Some concerns*	***-0.02*** ***, -0.07 to 0.03 (95% CI)***	***-0.03*** ***, -0.06 to 0.01 (95% CI)***	*No* (*p* = 0.89)	*The intervention was found to be effective overall. The study found no evidence for a difference in effect by socioeconomic status*

Intervention effect is bolded when there is evidence of an overall effect

*We reported the result as effective or not effective based on the estimates reported in the paper

† Adjusted difference in intervention and control difference in baseline to endline difference

‡ This result was not maintained over time with no effect reported at 21 months

§ Results not reported for grades 8–9

¶ The SEHER Mitra (SM) intervention was delivered by a lay counsellor and the teacher as SEHER Mitra (TSM) was delivered by a teacher

** There were 2 interventions, the interaction-based treatment and law and justice

## Discussion

Encouragingly, we find there is a growing number of RCTs of school-based violence prevention interventions, with 160 articles identified in this review. However, the evidence about equity of inclusion and equity of impact is limited. Most of 160 articles reported participant’s sex, but very few reported on other characteristics such as disability, sexuality, and gender identity. The current evidence on school violence prevention therefore does not meaningfully comment on inclusion of children who may be worse off, including minoritised children, making it challenging to understand how interventions affect the very subgroups who are more likely to experience violence.

Disappointingly, there is almost no good quality evidence on equity of impacts of school violence interventions. Only nineteen articles reporting on sixteen trials (11.9%) provided any evidence on equity of impact across different subgroups which may be more affected by violence. Trials that conducted a subgroup analysis also tended to score poorly on our equity criteria, and only 3 trials used appropriate statistical methods to explore subgroup differences. This is perhaps reflective of an evidence base that has to date rarely considered equity or how interventions impact subgroups. Across the studies, we find there was no clear pattern to effectiveness by subgroup. It is possible that studies were not adequately statistically powered to detect differences between subgroups, adding to the unclear pattern of the differences in intervention effectiveness observed in this review. It is less clear, then, if interventions equally benefit children more vulnerable to violence victimisation or reinforce inequities.

The limited evidence on equity within school violence trials we document in this paper is consistent with recent evidence in health or school trials where few sociodemographic characteristics are measured and reported [74] and subgroup analyses are not conducted, particularly in line with international guidelines [47, 48, 75–77]. Several factors could explain this including: limited study budgets and small sample sizes; research questions not concerned with equity; schools which are not demographically diverse; or participant decisions to not disclose sexuality, gender identity, wealth or other sociodemographic characteristics. Study design decisions (e.g. measurement of sociodemographic characteristics, sample sizes, and recruitment strategies for subgroups) by authors also play a large role in trial design and if, and how, to consider equity [78]. While collecting sensitive information, such as data on sexuality and gender identity, should be considered and planned for by context due to the high risk associated with discriminatory laws and societal stigmitisation [79], studies often do not describe justification for their exclusion. A recent scoping review found safe measurement can be possible if done in consultation with community experts [80] with careful consideration of the risks to participants.

The variability in subgroup effects by sex identified in this review is similar to recent evidence examining inequities in school-based dating and relationship violence and gender-based violence, that found evidence that interventions targeting dating violence perpetration reduction have a greater impact on boys [31]. For the Good Schools Toolkit, for instance, authors suggest lesser effects for girls could be related to a higher exposure to other forms of violence outside of schools and to their participation in the intervention which may be affected by competing pressures outside of school, including household chores [81]. It is also possible that interventions were not adequately addressing the gendered drivers of violence or engaging with the gendered nature of school contexts. Previous research has found interventions can have different impacts on subgroups due to the difference in mode of delivery – including some evidence that mixed sex delivery is less effective for boys compared to all-male groups [82] – and different mechanisms of impact [83]. Importantly, interventions have been found to increase inequities (intervention-generated inequalities) by only benefitting the least disadvantaged groups, highlighting a need to understand differential effects [35].

There are several limitations to this review. As the trials evaluated school-based interventions, the sample only includes children who attend school. Previous research suggests that children who are at a greater social disadvantage, and likely to experience higher levels of victimisation, are not always in school, which includes children with disabilities [84]. Due to the large number of systematic reviews focusing on different violence outcomes in schools, we designed an efficient search strategy to identify these systematic reviews and to sample our papers. The most recent systematic review included papers up to December 2023, but we will not have included papers published after this date. In addition, using systematic reviews to sample our papers relies on the search strategy of other review authors, meaning we may not be able to account for bias in database selection, inclusion criteria, and screening.

There are several implications of our findings that can guide future violence prevention research and implementation to build evidence on equity within violence work. While effective universal violence prevention interventions are undoubtedly helpful for some children, practitioners designing and delivering interventions may be able to maximise their benefit by developing specific additional content, or ways to deliver that content, to groups at higher risk. This will require an understanding of any differing needs (e.g. including multiple languages, adapted communication formats), differences in lived experiences, including representative materials (e.g. including a range of different sociodemographic groups in pictures or promotion materials), and training intervention facilitators in adjustments for different groups (e.g. disability-related adjustments). In the Fourth R intervention, for instance, facilitators and participating teachers discussed how the intervention materials could be adapted to account for varying class period lengths and student composition (e.g., mixed-sex, students with disabilities) [85]. Interventions should be designed to target different structural, institutional, community and interpersonal drivers of violence against children at high-risk of victimisation, including targeting the reduction in stigma and discrimination, and harmful gender norms and expectations [11, 86, 87].

To generate evidence on equity of intervention impacts, future trials could utilise our equity criteria to design equity-relevant school-based violence prevention trials, considering the sampling, analysis methods, recruitment, and intervention adaptations (Table 1). For instance, trials of school-based violence prevention interventions should consider powering trials to detect effects amongst children who are more likely to experience violence. To do this, researchers could consider oversampling or stratifying sampling to improve power, depending on the size of the subgroup of interest. This is particularly important to understand the variation in effect amongst different sociodemographic characteristics and to ensure the intervention improves outcomes for subgroups in addition to achieving ‘on average’ effects. The additional costs for a larger sample may be marginal and donors could consider utilising funds for increasing these as a cost-effective mechanism to generate increased evidence on equity. In addition, evaluating targeted interventions for children more likely to experience violence victimisation (e.g. children of minority sexualities) may be appropriate if sufficient sample sizes are difficult to achieve within universal interventions. Including a process evaluation alongside a trial may elucidate heterogeneity of intervention effects in addition or in lieu of subgroup analysis (if difficult to achieve).

## Conclusions

There has been increasing global attention to preventing violence against children, including the SDGs, WHO’s INSPIRE guidelines, and the 2024 Global Ministerial Conference on Ending Violence Against Children [88–90]. To meet or make progress towards targets set by 2030, and to achieve violence prevention at scale, we need evidence on how to prevent violence against all children, inclusive of minoritised children, or progress towards these goals and targets will only be met amongst the least marginalised children.

## Supplementary Information

Supplementary Material 1.

Supplementary Material 2.

## Data Availability

All data used in this article are publicly available.
