# Supplementary material for: A systematic review and equity analysis of school-based violence prevention interventions evaluated in randomised controlled trials
Source: BMC Public Health. 2026 Jan 28;26:681. doi: 10.1186/s12889-025-26142-1 (PMC12922353; doi:10.1186/s12889-025-26142-1)
Supplement: Supplementary file 2 — Supplementary Material 2. [file 12889_2025_26142_MOESM2_ESM.docx]

**Supplementary Materials**

**A systematic review and equity analysis of school-based violence prevention interventions evaluated in randomised controlled trials**

Emily Eldred, Karen Devries, Kate A Nelson, Anja Zinke-Allmang, Charles Opondo, Waliyah Mughis, Rizwana Mallick, Lena Morgon Banks and Amiya Bhatia

**Appendix contents**

[Appendix 1. PRISMA 2](#_Toc213832368)

[Appendix 2. Detailed equity criteria for assessing trial design of school-based violence prevention trials that conducted a subgroup analysis 3](#_Toc213832369)

[Appendix 3. Study characteristics of school-based violence prevention trials that included subgroup specific effect estimates 6](#_Toc213832370)

[Appendix 4. Risk of Bias (ROB) assessment of school-based violence prevention trials that included sub-group specific effect estimates^1^ 11](#_Toc213832371)

[Appendix 5. Search Terms & Databases 12](#_Toc213832372)

[Appendix 6. References 14](#_Toc213832373)

Appendix 1. PRISMA

7,256 articles identified through Medline, Cochrane, Embase, Global Health, PsycInfo and Web of Science database search

748 articles identified from included systematic reviews (n=29) in stage 1

2,102 duplicates excluded

5,154 articles screened title and abstract

5,124 excluded

30 articles screened at full text

511 articles screened at full text

**160 articles included at stage 2**

237 duplicated excluded

**351 excluded**

225 wrong study design (not an RCT)

1 outcomes not measured among school children

9 wrong population (not a school)

30 not a violence outcome

4 not able to access

16 not in English

48 thesis or dissertation

2 conference proceedings

16 book

**19 articles included at stage 3**

**3 excluded**

1 wrong study design (no RCT papers)

2 full text not in English

**141 excluded**

72 did not have a victimisation outcome

70 did not have empirical results for a subgroup effect estimate/test for heterogeneity with a comparator

2 additional reviews identified through professional networks

29 systematic reviews included at stage 1

**Stage 1:** identification of systematic reviews

**Stage 2:** identification of RCTs of school-based violence prevention interventions

**Stage 3:** identification of trials with subgroup analysis

Appendix 2. Detailed equity criteria for assessing trial design of school-based violence prevention trials that conducted a subgroup analysis

| **No.** | **Criteria** | **Definition of key terms** | **Explanation of criteria and scoring (0 or 1)** | **How to apply criteria** | **Illustrative example*** |
| --- | --- | --- | --- | --- | --- |
| 1 | Is the research question or hypothesis concerned with measuring equity or differences between groups? | (In)Equity: an inequality in health that is unjust, unfair and preventable (1)  (In)equality: used to ‘designate differences, variations, and disparities in the health achievements of individuals and groups’ (1)  Subgroup: “In randomized clinical trials, subgroup analyses evaluate the treatment effect (e.g., a hazard ratio [HR]) for a specific endpoint (e.g., overall survival) in subgroups of patients defined by baseline characteristics (e.g., age, gender, histology, and ethnicity)" (2) | Studies were given a score of 1 if they met any of the following criteria if the research question or hypothesis:   1. Included any of the following terms in relation to two subgroups: equity, inequity, inequality, difference.  or 2. Included testing intervention effects between two subgroups with a hypothesis about which group would benefit more/less from the intervention specified a priori. | (1) Search the key terms of ‘question’, ‘aim’, ‘objective’ or ‘hypothesis’  (2) If the research question is not found, read the abstract, introduction and methods of the paper to extract the research question  (3) Extract the research question and assess against criteria in detailed criteria column  (4) Score the criteria as 1 or 0 | ‘Whether there is a difference in the effects of the intervention by gender’ (3) |
| 2 | Was the subgroup analysis reported to be adequately powered? | Authors state that the subgroup analysis was powered to detect subgroup specific effects in at least one group | Studies were given a score of 1 if:   1. authors describe/state that the study was powered to detect differences for at least one subgroup | (1) Search the key terms ‘power’ and ‘sample size’  (2) If no results from searches, read the methods and the table footnotes in the main and subgroup tables  (3) Score the criteria as 1 or 0 | “We powered the trial to be able to detect a sex-specific effect size of 0·2 (difference in means/SD) with approximately 90% power” (4) |
| 3 | Did the paper report if subgroup analysis were pre-specified? | As above | Studies were given a score of 1 if the paper mentioned if:   1. subgroup analysis were specified a priori | (1) Search the key terms ‘pre-specified’ or ‘a priori’  (2) If no results from searches, read the methods and the table footnotes in the main and subgroup tables  (3) Score the criteria as 1 or 0 | Subgroup analysis was pre-specified in a statistical analysis plan or published protocol |
| 4 | Was the method of analysing and reporting the sub-group results appropriate? | Appropriate analysis: trials that reported an effect in different subgroups based on statistical significance without a test for heterogeneity may be misleading, as these estimates do not tell us about a difference between subgroups and are therefore not reporting on equity (5-7) | Studies were given a score of 1 if the method of analysing the sub-group was appropriate, based on:   1. reporting of a sub-group specific effect estimate using an interaction term and corresponding test for heterogeneity | (1) Read the methods and results  (2) Score the criteria as 1 or 0 | Not applicable |
| 5 | Was equity considered in sampling? | Sample: a randomised subset of the population of interest | Studies were given a score of 1 if the methods section included a description or reference to any of the following:   1. oversampling, or 2. stratified sampling, or 3. any other effort including for example, including a variation in the range of school sites (rural vs urban) | (1) Search for ‘sample’, ‘population’, or ‘participants’  (2) If no results from searches, read the methods sections and study participant description in results  (3) Score the criteria as 1 or 0 | ‘Separate sampling frames  were prepared for girls and boys of classes 6 and 7 after pooling the attendance rosters of all sections. From the sampling frames, 23 girls and 23 boys were selected randomly from each school for the BL survey’ (8) |
| 6 | Was equity considered in the design of survey implementation? | Survey: includes both the survey measures and/or the format of the survey | Studies were given a score of 1 if they included a description or reference to any of the following:   1. adaptations to informed consent procedures (e.g. witness, thumbprint), or 2. consent procedures for parents included oral explanations of study and reimbursement for travel costs, or 3. adjustments to the survey delivery and questions (e.g. multiple languages were used to administer the survey that were relevant to the population), or 4. training was provided to researchers on reasonable adjustments (e.g. for disability), or 5. measures about inequities were included in the survey (e.g. measurement of gender equity), or 6. any other similar adaptations made, in discussion with co-authors | (1) Read ‘measures’ or ‘study design’ section of methods  (2) Score the criteria as 1 or 0 | ‘Reasonable interview adjustments for students with sight, hearing, or other functional difficulties’ (9) |
| 7 | Was equity considered in study response or referral plans? | Response/referral plan: a protocol or procedure for receiving disclosures of violence or severe distress during the research data collection. An inclusive referral plan for cases of disclosures of violence is an essential to violence research (10) | Studies were given a score of 1 if they included a description or reference to any of the following:   1. adaptations to procedures for minoritised groups that are context specific. For example, children with disabilities (e.g. consultation with social workers about capacity for receiving disclosures of children with different impairments, including sign language) | (1) Search for ‘refer’ or ‘response’  (2) If no results from searches, read the methods sections  (3) Score the criteria as 1 or 0 | A referral or response plan is mentioned to include adaptations for children with disabilities (e.g. non-verbal communication pathways for disclosures) |
| 8 | Was there any adaptation to intervention content for subgroups? | Intervention: the intervention being tested in the randomised controlled trial  Adaptation: flexibility in the content and delivery to accommodate differences in the population | Studies were given a score of 1 if they included a description or reference to any of the following:   1. any form of adaptation to intervention materials (e.g. audio-visual, braille), teachers or implementers trained in reasonable adjustments, or 2. representative intervention material pictures and stories, or 3. testing of intervention in different sociodemographic groups, or 4. different languages used in intervention materials, or 5. any other similar adaptations made to improve access to and/or impact of intervention for subgroups, in discussion with co-authors | (1) Read intervention description section in introduction or methods  (2) Score the criteria as 1 or 0 | ‘The facilitator and participating teachers role-played select program materials and exercises and discussed how materials might be adapted to account for varying class period lengths, student composition (e.g., mixed-sex, IEP students), and so on. IEP is individualized education plans (disability)’ (11) |
| To develop criteria, we drew on existing reporting checklists, including CONSORT-Equity, REP-EQUITY and PROGRESS-PLUS, international guidelines on subgroup analysis, and expertise within the authorship team, and adapted these for school-based violence prevention trials (5-7, 12-14). We reviewed what was reported in the papers only.  *Examples of studies which would score 1 are either taken from papers in this review or are illustrative | | | | | |

Appendix 3. Study characteristics of school-based violence prevention trials that included subgroup specific effect estimates

|  | **Trial citation (lead author; year)** | **Description of intervention (name; description; location; school type)** | **Trial characteristics (design; violence victimisation outcome(s); registration)** | **Sociodemographic characteristics measured** | **Sociodemographic characteristic included in sub-group analysis** | **Equity assessment score*** |
| --- | --- | --- | --- | --- | --- | --- |
| 1 | Achyut 2017 (Viet Nam) | **Name:**  Gender Equity Movement in Schools (GEMS)  **Description:** multiple activities with teachers, students and parents including orientation, curriculum with students and school-based campaigns. Adapted for Viet Nam from Indian programme pilot  **Location:** Viet Nam  **School type:** secondary | **Description:** randomised controlled trial  **Violence victimisation outcome:** Any violence victimisation by peers in the last semester; any violence victimisation by teachers in the last 3 semesters  **Registration:** not registered | Sex; parents’ education; income status | Sex | Medium (4/8) |
| 2 | Achyut 2017 (India) | **Name:**  Gender Equity Movement in Schools (GEMS)  **Description:** multiple activities with teachers, students and parents including orientation, curriculum with students and school-based campaigns  **Location:** India  **School type:** secondary | **Description:** randomised controlled trial  **Violence victimisation outcome:** Any violence victimisation by teacher or peer in the last 3 months  **Registration:** not registered | Sex; parents’ education; income status | Sex | Medium (4/8) |
| 3 | Bonell 2018 | **Name:** Learning Together  **Description:** a whole-school intervention including restorative practice aiming to resolve and prevent conflicts in school and social and emotional education  **Location:** UK  **School type:** secondary | **Description:** cluster randomised controlled trial  **Violence victimisation outcome:**  experience of bullying victimisation in the past 3 months  **Registration:** ISRCTN registry (10751359) | Race/ethnicity; sex; income status; religion; family structure; parents’ employment; parents’ housing | Sex; socioeconomic status | Low (2/8) |
| 4 | Cissner 2014 | **Name:** Fourth R  **Description:** a 21-lesson skills-based curriculum for adolescents, aiming to develop healthy decision-making in relationships, sexuality and drug and alcohol use  **Location:** USA  **School type:** secondary | **Description:** cluster randomised controlled trial  **Victimisation outcome:** dating violence victimisation; peer victimisation; sexual harassment/assault victimisation  **Registration:** not registered | Disability; race/ethnicity; sexuality; sex; income status; family structure | Sex | Low (1/8) |
| 5 | Coker 2017 and 2020 | **Name:** Green Dot  **Description:** a bystander intervention with training for students and teachers to deliver school-wide presentations to reduce sexual violence  **Location:** USA  **School type:** secondary | **Description:** cluster randomised controlled trial  **Victimisation outcome:** sexual violence victimisation  **Registration:** ClinicalTrials.gov ID NCT01878097 | Race/ethnicity; sexuality; sex; income status | Sex; sexuality | None reported (both papers) |
| 6 | Devries 2017 and 2018 | **Name:** Good Schools Toolkit  **Description:** a complex behavioural intervention aiming to target a reduction in violence at the school level  **Location:** Uganda  **School type:** primary | **Description:** cluster randomised controlled trial  **Victimisation outcome:** any violence from teachers or school staff in the past week  **Registration:** clinicaltrials.gov IDNCT01678846 | Disability and functional limitation; sex; income status | Sex; disability and functional limitation | High (6/8) (both papers) |
| 7 | Gradinger 2015 | **Name:**  ViSC Social Competence Program  **Description:** a primary prevention program to reduce bullying and foster social and intercultural competencies within school though teacher training and a student curriculum  **Location:** Austria  **School type:** secondary | **Description:** cluster randomised controlled trial  **Victimisation outcome:**  cyberbullying victimisation in the past 2 months  **Registration:** not registered | Ethnicity; sex; income status | Sex | None reported |
| 8 | Gusmoes 2018 | **Name:** #Tamojunto prevention program  **Description:** Curriculum for students taught by teachers, with 12 lessons on attitudes and knowledge of drugs, interpersonal skills, and personal skills  **Location:** Brazil  **School type:** primary | **Description:** cluster randomised controlled trial  **Victimisation outcome:**  physical violence victimisation in the past 30 days; bullying victimisation in the past 30 days  **Registration:** Brazilian Register of Clinical Trials (REBEC): RBR-4 mnv5 g | Sex; income status | Sex | None reported |
| 9 | Jemmott 2018 | **Name:**  Let Us Protect Our Future  **Description:** curriculum-based intervention to reduce risky sexual behaviours with content on gender and rape myths  **Location:** South Africa  **School type:** secondary | **Description:** cluster randomised controlled trial  **Victimisation outcome:** experiences of forced sex  **Registration:** ClinicalTrials.gov Identifier: NCT00559403 | Ethnicity; sex | Sex | Low (1/8) |
| 10 | Karna 2011 and 2013; Williford 2013 | **Name:** KiVa  **Description:** a curriculum-based intervention delivered by teachers to raise awareness of the role that the children can play in maintaining bullying, (b) increase empathy toward victims, and (c) promote children’s strategies of supporting the victim  **Location:** Finland  **School type:** Mixed | **Description:** cluster randomised controlled trial  **Victimisation outcome:** (1) self-reported bullying victimisation; (2) cybervictimisation  **Registration:** not registered | Sex; ethnicity; second language | Sex | None reported |
| 11 | Karmaliani 2020 | **Name:** Right to Play  **Description:** a play-based programme aiming to influence academic achievement and substance misuse.  **Location:** Pakistan  **School type:** primary | **Description:** cluster randomised controlled trial  **Victimisation outcome:**  peer victimisation in the last 4 weeks  **Registration:** ClinicalTrials.gov ID NCT03448523 | Sex; income status | Sex | Medium (3/8) |
| 12 | Ostrov 2015 | **Name:** Early Childhood Friendship Project (ECFP)  **Description:** a classroom-based  intervention program for early childhood to reduce physical and relational forms of aggression and victimization  **Location:** USA  **School type:** primary | **Description:** cluster randomised controlled trial  **Victimisation outcome:**  physical and relational victimisation  **Registration:** not registered | Race/ethnicity; sex | Sex | None reported |
| 13 | Peskin 2014 | **Name:**  It’s Your Game…Keep it Real (IYG)  **Description:** classroom and computer-based activities for students to support healthy relationships  **Location:** USA  **School type:** secondary | **Description:** cluster randomised controlled trial  **Victimisation outcome:** physical dating violence victimisation; emotional dating violence victimisation  **Registration:** ClinicalTrials.gov, NCT03482687 | Sex; race/ethnicity | Sex; race/ethnicity | Low (1/8) |
| 14 | Shinde 2018 | **Name:** Strengthening Evidence base on scHool-based intErventions for pRomoting adolescent health programme (SEHER)  **Description:** a whole school health promotion programme, including whole school, class-targeted and individual focused components to improve health and wellbeing within schools  **Location:** India  **School type:** secondary | **Description:** cluster randomised controlled trial  **Victimisation outcome:**  violence victimisation in the past 6 months  **Registration:** ClinicalTrials.gov, NCT02484014 | Sex; social class/caste | Sex | Medium (3/8) |
| 15 | Sorrentino 2018 | **Name:**  Tabby Improved Prevention and Intervention Program (TIPIP)  **Description:** program has four key components: teacher training; school conferences with parents; in-class activities; online materials for all  **Location:** Italy  **School type: s**econdary | **Description:** randomised controlled trial  **Victimisation outcome:** cybervictimisation in the past 6 months  **Registration:** not registered | Sex | Sex | None reported |
| 16 | Taylor 2010 | **Name:** Unnamed interventions  **Description:** Interaction-based curriculum and a law and justice curriculum taught to separate groups with 5 lessons across 5 weeks  **Location:** USA  **School type:** Secondary | **Description:** cluster randomised controlled trial  **Victimisation outcome:**  any violence victimisation from peers  **Registration:** not registered | Race/ethnicity; sex | Sex | Medium (2/8) |
| * Scoring criteria for the equity assessment are as follows: scores were assigned to each equity item to give each study an overall score from ‘none reported’ (0 items), ‘low’ (1-2 items), ‘medium’ (3-5 items), or ‘high’ (6-8 items). | | | | | | |

Appendix 4. Risk of Bias (ROB) assessment of school-based violence prevention trials that included sub-group specific effect estimates^1^


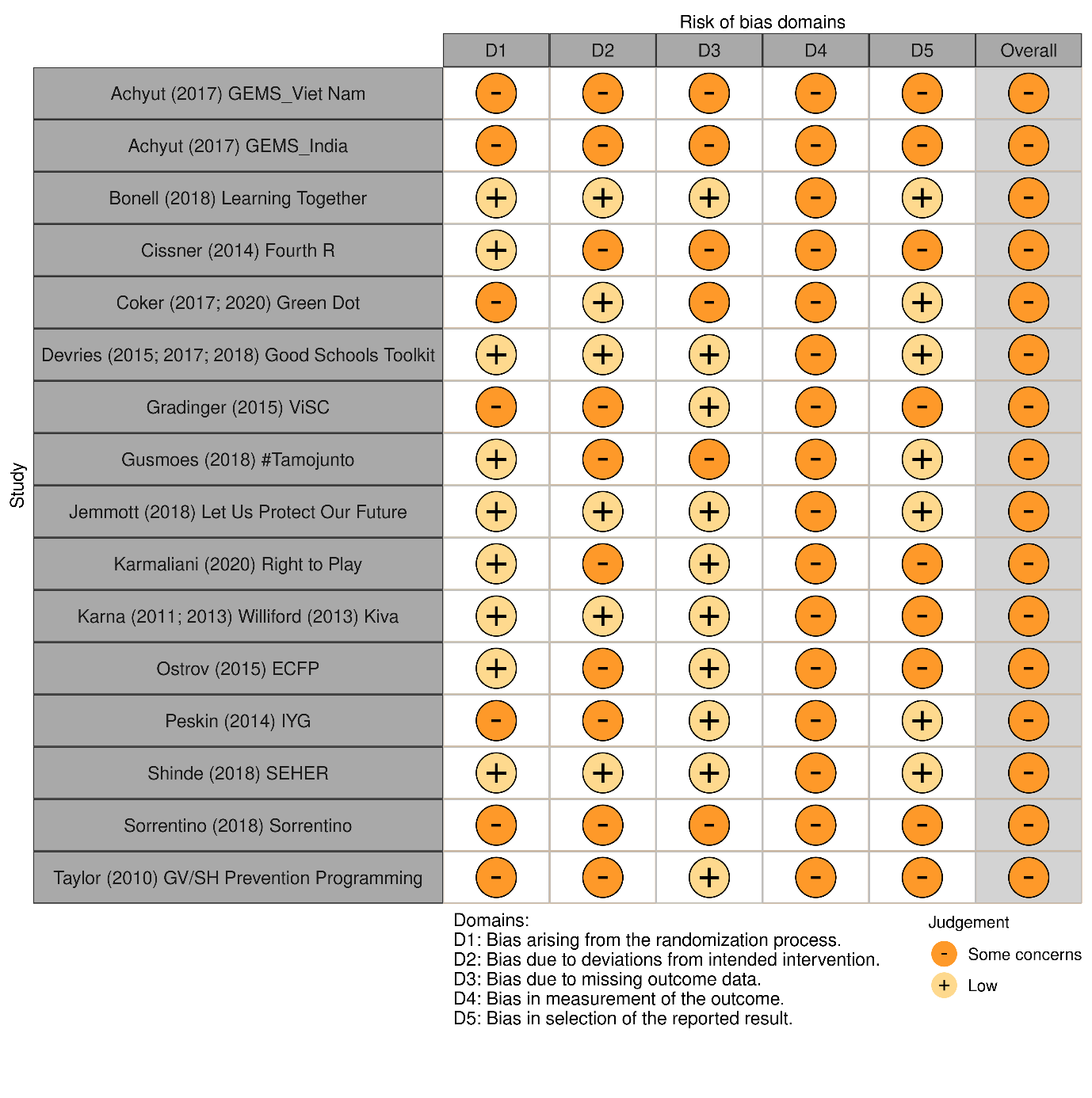

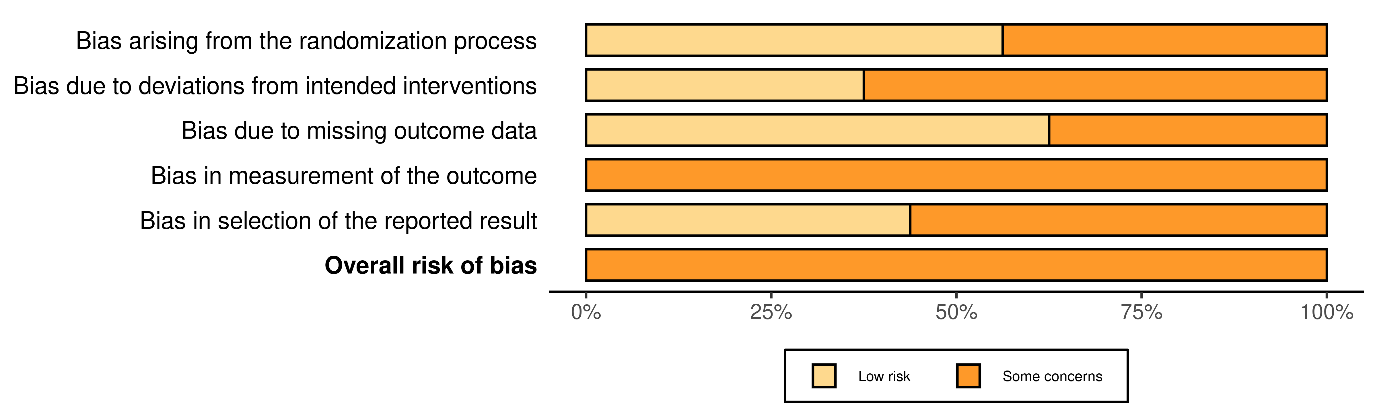


**^1^**These plots were generated using the Risk-of-bias VISualization (robvis) tool, available here: McGuinness, LA, Higgins, JPT. Risk-of-bias VISualization (robvis): An R package and Shiny web app for visualizing risk-of-bias assessments. Res Syn Meth. 2020; 1- 7. https://doi.org/10.1002/jrsm.1411

Appendix 5. Search Terms & Databases

Databases to search: MEDLINE, EMBASE, PsycINFO, Global Health, and Web of Science (Science and Social Science Citation Index); Cochrane Library

OVID

Medline (1699), Embase (2375)

1. Randomized Controlled Trial/ or Early Intervention, Educational/

2. (prevention or preventing or reduce or reducing or intervention or programme or program or project* or initiative or evaluation or evaluate or trial or randomi#ed control trial or effectiveness). ti,ab,kw.

3. 1 or 2

4. Child/ or Adolescent/ or Young Adult/ or Infant/ or Infant, Newborn/ or Students/ or Child, Preschool/

5. (child* or adolescen* or boy$1 or boyhood or girl* or teen* or preteen* or pubescen* or prepubescen* or youth* or juvenile* or preteen* or pre teen* or young people* or young person* or early adult* or young adult* or infan* or baby or babies or school* or pupil* or student* or nursery or preschool* or pre school* or underage or minor or partner* or spouse* or peer* or boyfriend* or boy friend* or girlfriend* or girl friend or acquaintance* or non stranger* or nonstranger* or agemate or age mate).ti,ab,kw.

6. 4 OR 5

7. Schools, Nursery/

8. (School* or classroom or learning institution or educational institution or school-based or education). ti,ab,kw.

9. 7 OR 8

10. Violence/ or Physical Abuse/ or Rape/ or Adverse Childhood Experiences/ or Child Abuse/

11. (violence or violent or aggression or maltreatment or maltreat* or abuse or corporal punish* or discipline or spanking or caning or peer violence or peer relationships or bully* or bullied or anti bully* or bully victim or cyberbull* or cybervictim* or intimate partner violence or IPV or gender based violence or GBV or dating or teen relationships or violence against children or VAC or coercion or defilement or rape$1 or rapist or incest or polyvictim* or pedophil* or peadophil* or sexual assault or restraining or exclusion or neglect* or psychosocial). ti,ab,kw.

12. sex* adj2 (violen* OR abus* OR assault* OR attack* OR aggressi* OR coerc* OR maltreat* OR victim* OR re victim* OR offence* OR offense* OR molest* OR harass* OR exploit*).ti,ab,kw

13. phys* adj2 (violen* OR abus* OR assault* OR attack* OR aggressi* OR coerc* OR maltreat* OR victim*).ti,ab,kw.

14. ((emotion* OR psychologic* OR mental) adj2 (violen* OR abus* OR maltreat*)).ti,ab,kw.

15. ((gender or peer) adj2 (violen* OR abus* OR assault* OR attack* OR aggressi* OR coerc* OR maltreat* OR victim* OR harass*)).ti,ab,kw.

16. ((intimate partner OR domestic partner OR partner* OR relationship* OR spouse* OR boyfriend* OR boy friend* OR girlfriend* OR girl friend OR date OR dating OR acquaintance* OR non stranger* OR nonstranger*) adj2 (violen* OR abus* OR assault* OR attack* OR aggressi* OR coerc* OR maltreat* OR victim*)).ti,ab,kw.

17. ((witness* OR expos*) adj2 (violen* OR abus* OR assault* OR attack* OR aggressi* OR coerc* OR maltreat* OR harass*)).ti,ab,kw.

18. 10 or 11 or 12 or 13 or 14 or 15 or 16 or 17

19. Review/ or Systematic Review/ or meta-analysis/

20. (Review* or systematic review or meta-analy* or meta analy* or synthesis) or (((comprehensive* or integrative or systematic*) adj3 (bibliographic* or review* or literature)) or (meta-analy* or metaanaly* or "research synthesis" or ((information or data) adj3 synthesis) or (data adj2 extract*))).ti,ab,kw.

21. 19 or 20

22. 3 and 6 and 9 and 18 and 21

23 limit 22 to last 5 years

Appendix 6. References

1. Kawachi I, Subramanian SV, Almeida-Filho N. A glossary for health inequalities. Journal of Epidemiology and Community Health. 2002;56:647-52.

2. Barraclough H, Govindan R. Biostatistics Primer: What a Clinician Ought to Know: Subgroup Analyses. Journal of Thoracic Oncology. 2010;5.

3. Taylor B, Stein N, Burden F. The effects of gender violence/ harassment prevention programming in middle schools: a randomized experimental evaluation. Violence Vict. 2010;25(2):202-23.

4. Shinde S, Weiss HA, Varghese B, Khandeparkar P, Pereira B, Sharma A, et al. Promoting school climate and health outcomes with the SEHER multi-component secondary school intervention in Bihar, India: a cluster-randomised controlled trial. Lancet. 2018;392(10163):2465-77.

5. European Medicines Agency. Guideline on the investigation of subgroups in confirmatory clinical trials. 2019.

6. Wang R, Lagakos SW, Ware JH, Hunter DJ, Drazen JM. Statistics in Medicine — Reporting of Subgroup Analyses in Clinical Trials. The New England Journal of Medicine. 2007;357.

7. Higgins JPT, Li T, Deeks JJ. Chapter 6: Choosing effect measures and computing estimates of effect [last updated August 2023]. In: Higgins JPT, Thomas J, Chandler J, Cumpston M, Li T, Page MJ, et al., editors. Cochrane Handbook for Systematic Reviews of Interventions version 65. Cochrane2024.

8. Achyut.P, Bhatla N., Kumar U., Verma H., Bhattacharya S., Singh G, et al. Changing Course: Implementation and Evaluation of the Gender Equity Movement in Schools (GEMS) program in specific sites –Vietnam, India and Bangladesh. New Delhi: International Center for Research on Women; 2017.

9. Devries K, Kuper H, Knight L, Allen E, Kyegombe N, Banks LM, et al. Reducing Physical Violence Toward Primary School Students With Disabilities. J Adolesc Health. 2018;62(3):303-10.

10. Bhatia A, Zinke-Allmang A, Bangirana CA, Nakuti J, Amollo M, Mirembe AF, et al. Putting children's safety at the heart of violence research. Nat Med. 2024;30(10):2721-4.

11. Cissner AB, Ayoub LH. Building Healthy Teen Relationships. 2014.

12. Welch V, Petticrew M, Petkovic J, Moher D, Waters E, White H, et al. Extending the PRISMA statement to equity-focused systematic reviews (PRISMA-E 2012): explanation and elaboration. Int J Equity Health. 2015;14:92.

13. Mbuagbaw L, Aves T, Shea B, Jull J, Welch V, Taljaard M, et al. Considerations and guidance in designing equity-relevant clinical trials. Int J Equity Health. 2017;16(1):93.

14. Retzer A, Ciytak B, Khatsuria F, El-Awaisi J, Harris IM, Chapman L, et al. A toolkit for capturing a representative and equitable sample in health research. Nat Med. 2023;29(12):3259-67.
